# Comparison of axial length using a new swept-source optical coherence tomography-based biometer - ARGOS with partial coherence interferometry- based biometer -IOLMaster among school children

**DOI:** 10.1371/journal.pone.0209356

**Published:** 2018-12-27

**Authors:** Jameel Rizwana Hussaindeen, Ephrame G. Mariam, Sushil Arunachalam, Ramakrishnan Bhavatharini, Aparna Gopalakrishnan, Anuradha Narayanan, Sumita Agarkar, Viswanathan Sivaraman

**Affiliations:** 1 Myopia Clinic, Srimathi Sundari Subramanian Department of Visual Psychophysics, Unit of Medical Research Foundation, Sankara Nethralaya, Nungambakkam, Chennai, India; 2 The Sankara Nethralaya Academy, Unit of Medical Research Foundation, Sankara Nethralaya, Nungambakkam, Chennai, India; 3 Elite School of Optometry, Unit of Medical Research Foundation, St. Thomas Mount, Chennai, India; 4 Pediatric Ophthalmology Department, Sankara Nethralaya, Unit of Medical Research Foundation, Nungambakkam, Chennai, India; Universidad de Monterrey Division de Ciencias de la Salud, MEXICO

## Abstract

**Purpose:**

To compare the axial length measurements obtained by a new swept source optical coherence tomography based biometer-ARGOS with partial coherence interferometry based biometer -IOL master in school children between the ages of 11–17.

**Methods:**

A prospective, cross-sectional, device comparison study was conducted in a school vision screening program comparing the axial length (AL) and corneal curvature (K) measurements obtained by two biometers- ARGOS and IOL master. Children with 6/9 vision or better, without any ocular abnormalities were included in the study. Two trained optometrists performed the measurements and were masked for the outcome measures.

**Results:**

The sample size was 188 with a mean(SD) age of 13.88±1.69 years, of which 101 were boys. The mean (SD) AL was 23.94± 1.01 mm with Argos and 23.83 ± 1.03 mm with IOL Master (paired t-test, p>0.05). The mean K was 43.62D±1.59 with Argos and 43.64D±1.61 with IOL master (paired t-test, p>0.05). There was a strong positive correlation between the biometers for AL (r = 1.00, p<0.0001) and K (r = 0.99, p<0.0001). The mean difference in axial length between the two biometers was 0.11± 0.04 mm and the limits of agreement were between -0.02 to -0.19. The mean difference in corneal curvature was 0.02±0.15D and the limits of agreement were between -0.28 to 0.32D.

**Conclusion:**

Axial length measurements using SS-OCT and PCI based biometers were in agreement and comparable among children between the ages of 11 to 17.

## Introduction

Over 12.8 million (0.96%) in the age range of 5–15 years are affected by inadequately corrected refractive error around the globe, with the highest prevalence reported in developed urban areas in south-east Asia and in China [1].In both rural and urban India, refractive error is the leading cause of preventable visual impairment among school children aged between 7 and 15 [2]. Ocular biometry parameters play the most important role in the process of emmetropization and refractive errors are considered as a failure in the compensation of biometry parameters during this process. Ocular components not only play a vital role in the determination of presence or absence of refractive errors but also in the magnitude. Previous studies have reported high correlation (0.77 to 0.89) [3,4] between axial length and refractive error, and it is understandable that accurate measure of axial length and other ocular biometric parameters among children are vital to identify potential risk factors in the onset and progression of refractive errors.

IOL Master (version 5; Carl Zeiss, Germany), is a non-invasive optical biometer that uses partial coherence interferometry (PCI) with a wavelength of 780 nm to measure axial length and is the current gold standard non-contact ocular biometer.[5] The Argos (Suntec, Inc., Aichi, Japan) is a new non-invasive optical biometer that uses swept-source optical coherence tomography (SS-OCT) with a wavelength of 1050 nm.[6] Axial lengths measured with Argos are referenced from the corneal surface to the retinal pigment epithelium using refractive indexes that correspond to each tissue (cornea-1.374; aqueous humor-1.336; lens-1.410, vitreous humor-1.336). The optical distances are converted to geometric distances based on the measurements across each surface with the help of an automatic algorithm [6]. SS-OCT improves tissue penetration because of the narrow-bandwidth (20 nm) of the wavelength of light used, resulting in improved image quality [7, 8]. There are few studies that have compared the agreement and the reliability of the new SS OCT biometer with IOL Master,[7,9,10,11] but no studies have looked at the agreement among pediatric population. Hence the purpose of this study was to compare major ocular biometry parameters namely axial length (AL) and mean anterior corneal curvature (K) measurements between the two biometers among school children.

## Methodology

**Fig 1:**
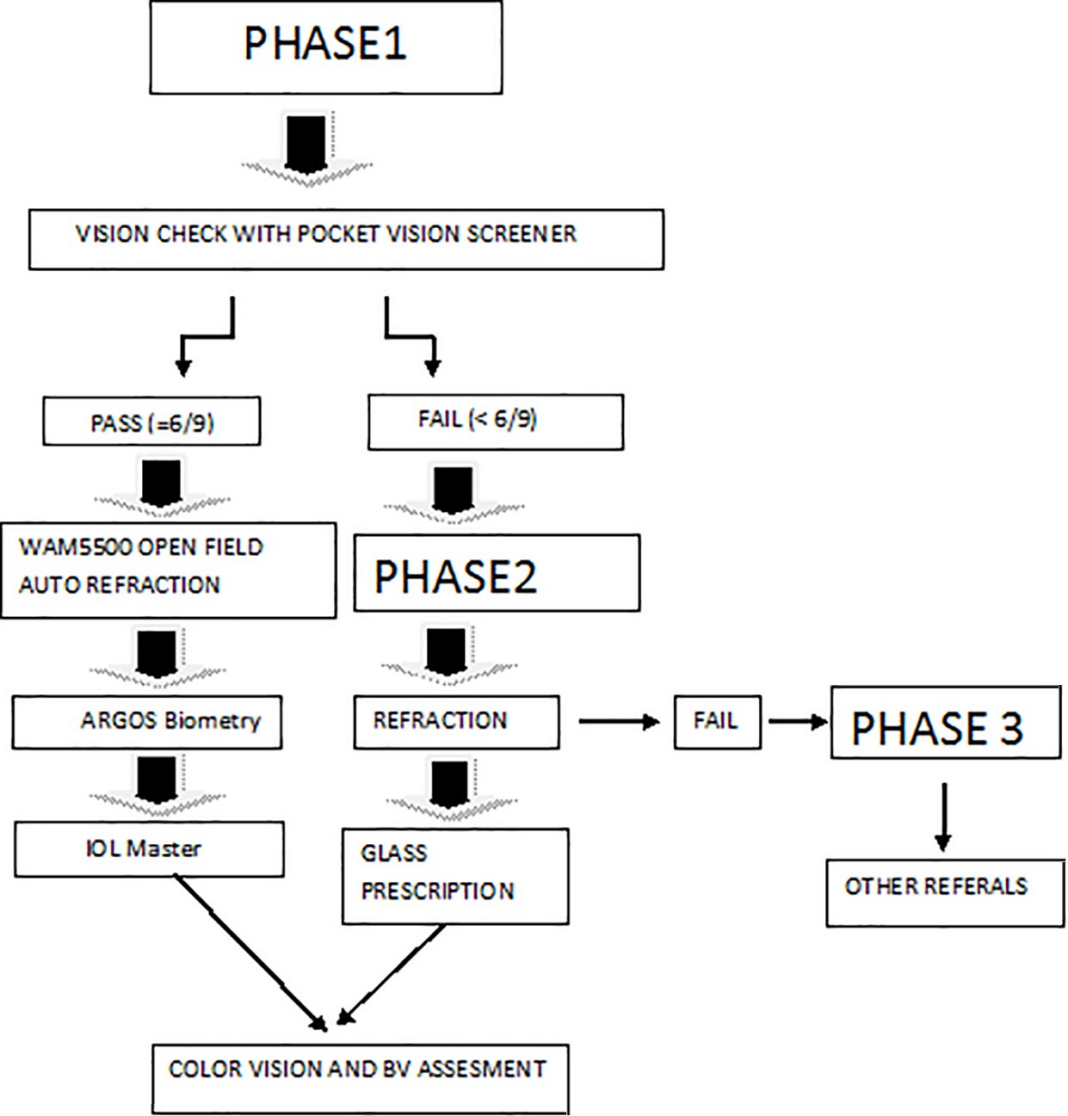
Flow chart of the school vision screening process.

The study was conducted as part of the School vision screening program of Elite school of Optometry and the present study aimed at profiling the normative data on biometry parameters and their correlation with refractive error measurements among children in Tamil Nadu, India. As the study was part of the large community vision screening program for schools in Kanchipuram district, permission to conduct the screening in schools was obtained from the Directorate of education for the district. This approval was then submitted to the school administration and a written informed consent detailing the procedures and process was obtained. The informed consent had the option for the parent to opt out of the study. The school then sent circular to the parents about the vision screening, and the investigators addressed the teachers through an awareness talk detailing the process and procedures. Parents were given an option to clarify doubts from the study investigators on the day of vision screening. This was then followed by the actual deployment of the project, and all the measurements were non-invasive. Oral assent was obtained from the children by explaining the procedure from the assent form and asking for their willingness to participate prior to conducting the tests. The Ethics committee and the Institutional Review Board of Vision Research Foundation approved this study protocol (Study code: 639-2017-p). The School vision screening protocol is depicted in Fig 1.

All children first underwent vision testing using a pocket vision screener [12] along with their habitual correction if any. Children with visual acuity of 6/9 or better in both the eyes next underwent refraction using WAM-5500 open field auto refractometer, followed by axial length and corneal curvature measurements using both IOL master and ARGOS. The ocular biometry measurements were performed by two trained and masked optometrists. The instruments were calibrated at the beginning of the screening every day as per manufacturer’s guidelines.

Children with best corrected visual acuity of 6/9 or better were included in the study and those who had any ocular abnormalities, developmental delay and uncooperative children were excluded from the study. The classification of refractive errors for the study was based on non cycloplegic open field autorefraction. Definitions of refractive errors were as follows:

Myopia: Spherical refraction of ≤-0.50D

Hyperopia: Spherical refraction of >+0.50D

Astigmatism: Cylindrical refraction of ≤-0.50D (negative cylinder form)

Emmetropia: Spherical refraction of +0.50 to -0.25D and cylindrical refraction of ≥-0.50D

Sample size calculation was based on the standardized difference and agreement limits [13] was done using Higashiyama et al’s [11] study, and was found to be 164.

## Statistical analysis

Data entry was done using Microsoft Excel spreadsheet and statistical analysis was performed using Medcalc for windows, Version 18.2.1. The normality of the numerical values was evaluated using the Shapiro-Wilk test. A paired t-test was used to compare the right and left eye axial lengths (ALs). Pearson’s product-moment correlation coefficient was used to analyze the correlation between the ALs and K acquired with the two biometers. The Bland-Altman limits-of Agreement (LoA) method [14] was used to assess the agreement in axial length and the mean anterior corneal curvature measurements between the two biometers.

## Results

The overall sample size was 188 subjects (376 eyes) of which 101 were boys and the mean (SD) age of the sample was 13.88± 1.69 years. Paired t-test between the two eyes showed no statistically significant difference (p = 0.83 for axl ARGOS and p = 0.90 for axl IOL Master) for both the biometer measurements. Hence the values of right eye alone were included for analysis. Normality of the parameters were tested using Shapiro-Wilk test and the distributions were normal (p = 0.13; 0.20).

The refractive error distribution of the overall sample and the refractive error range is shown in Table 1.

**Table 1:** Distribution of refractive error in the sample.

Refractive error	N = 188	Range
Myopia	90 (47.87%)	-0.50 to -9.68D
Hyperopia	26 (13.82%)	0.75 to 4.39D
Astigmatism (both simple and mixed)	95 (50.53%)	-0.50 to -3.98D
Emmetropia	44 (23.40%)	-0.25 to +0.50D

**Fig 2:**
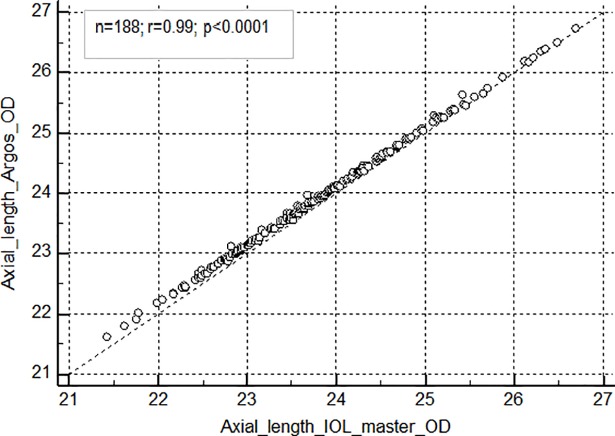
Correlation of axial length between Argos and IOL master biometers. AL-Axial length; OD–Right eye.

**Fig 3:**
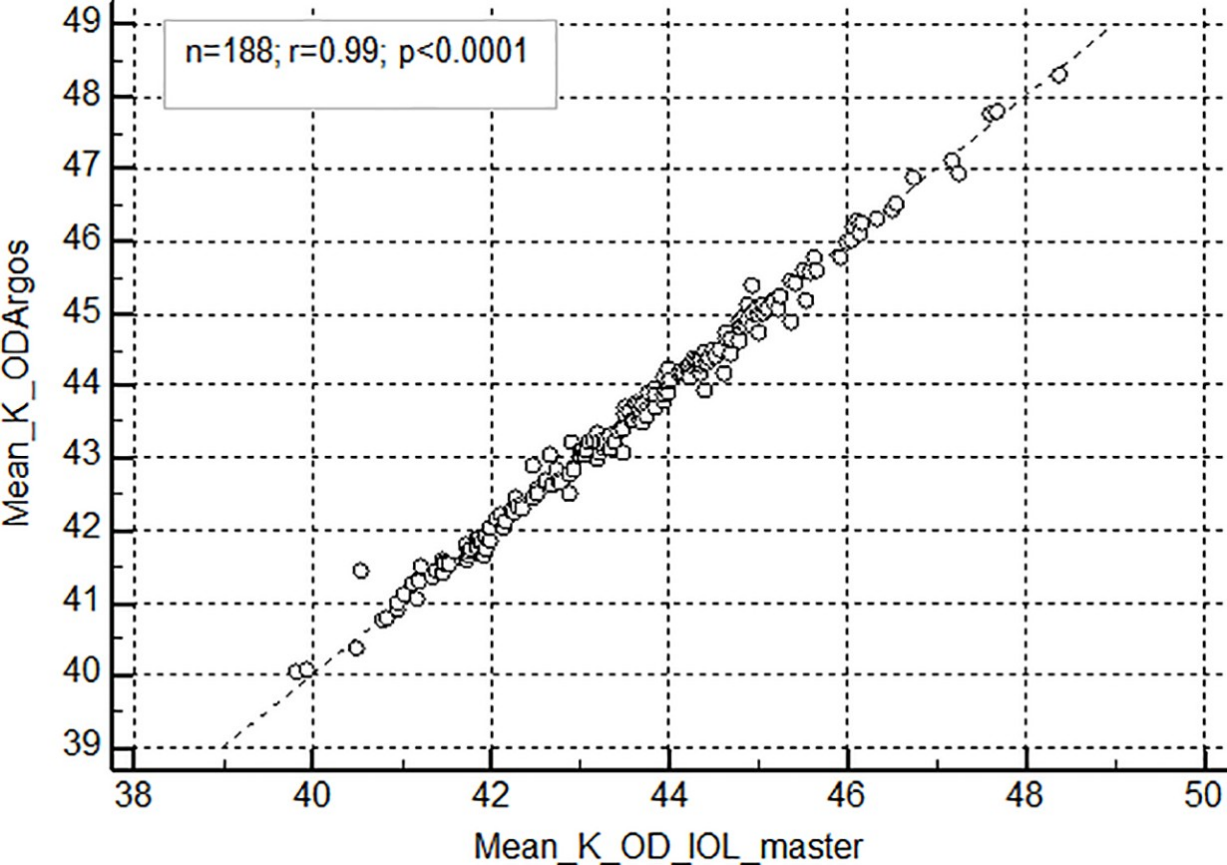
Correlation of Mean Corneal curvature (in Dioptres) between Argos and IOL master. K- Corneal curvature in Dioptres; OD–Right eye.

The mean (SD) AL was 23.93± 1.02 mm and 23.82 ± 1.05 mm with Argos and IOL Master respectively. There was a strong positive correlation between the biometers for both axial length (r = 0.99, 95% CI for r: 0.9992 to 0.9996; p<0.0001) (Fig 2) and corneal curvature measurements (Fig 3) (r = 0.99, 95% CI for r: 0.9940 to 0.9966; p<0.0001).

**Fig 4:**
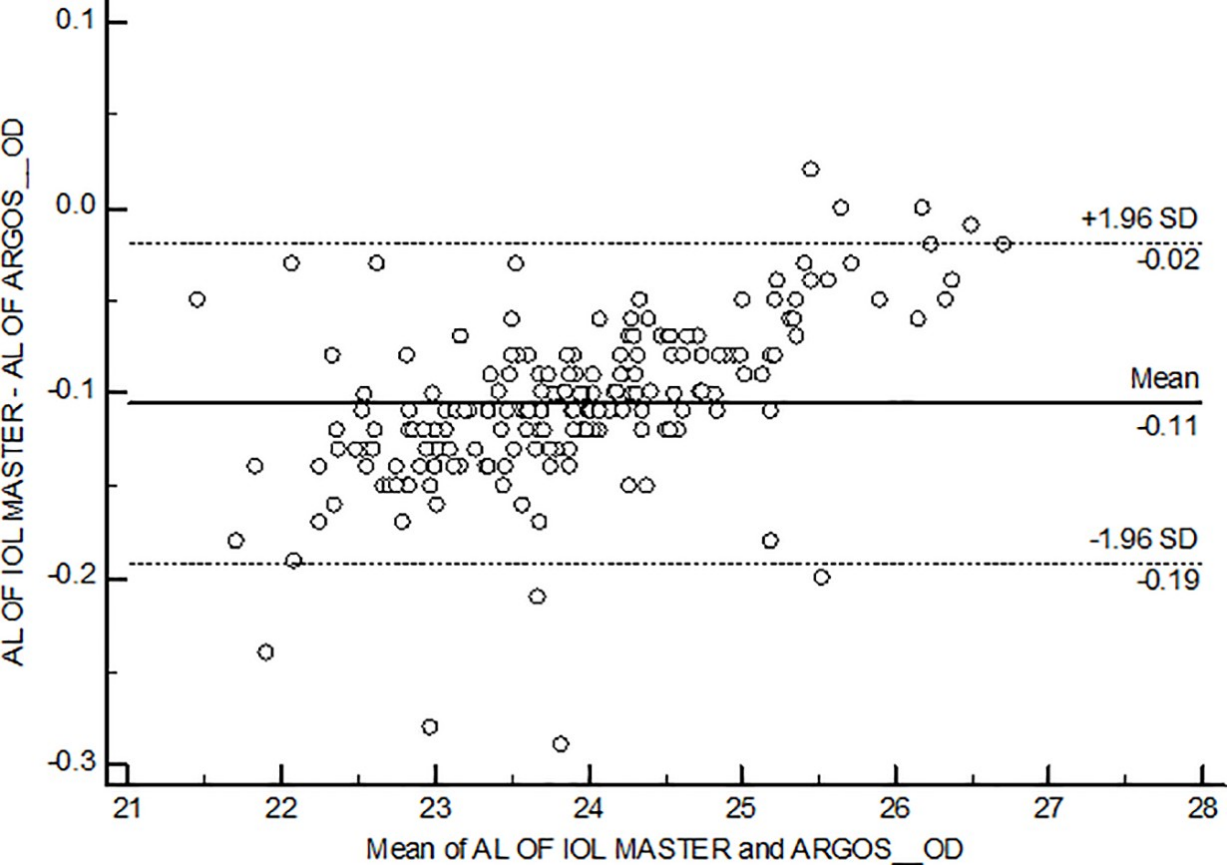
Bland-Altman plot for agreement between axial length using Argos and IOL master biometers. AL-Axial length; OD–Right eye.

**Fig 5:**
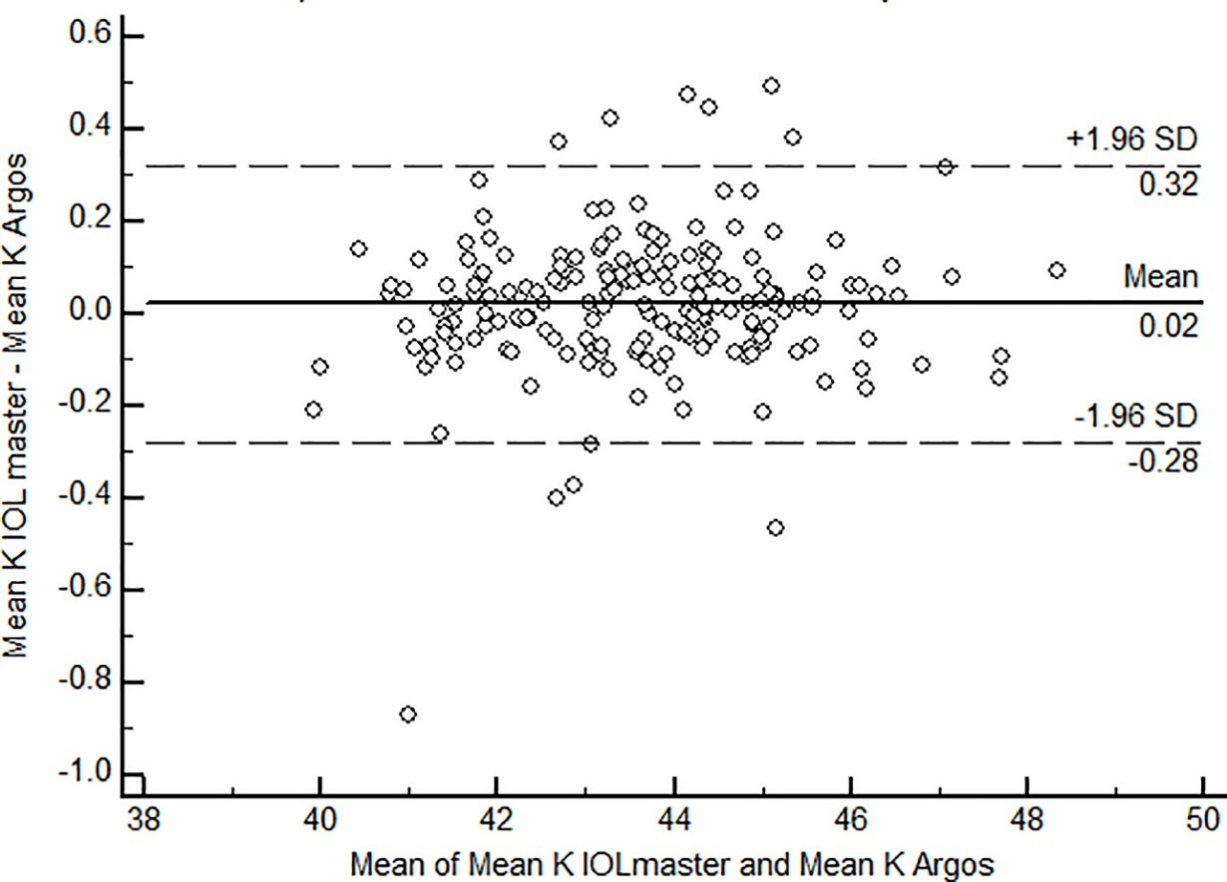
Bland-Altman plot of mean corneal curvature for measurements using Argos and IOL master biometers. K- Corneal curvature in Dioptres.

Agreement between the two biometers using the Bland-Altman plot for axial length and mean corneal curvature is shown in Fig 4 and Fig 5. The mean AL difference was 0.11± 0.05 mm with limits of agreement ranging between -0.02 to -0.19. The mean corneal curvature difference was 0.02 D and the limits of agreement were -0.28 to 0.32.

The axial length measurements were further divided into short (<23.27mm), Intermediate (23.27–24.03mm) and long (≥24.04) [11]

**Fig 6:**
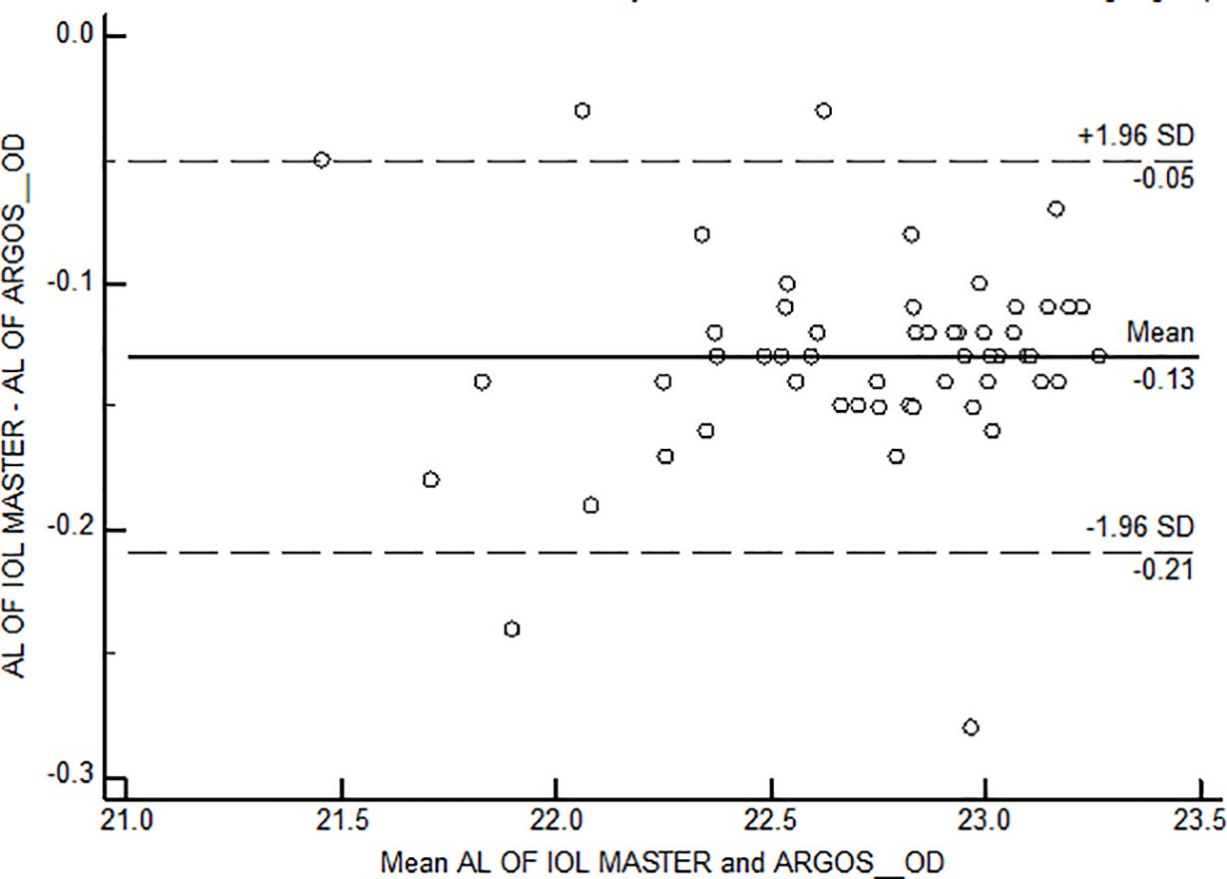
Bland Altman Plot for agreement between axial length measurements among short axial length group. AL-Axial length; OD–Right eye.

**Fig 7:**
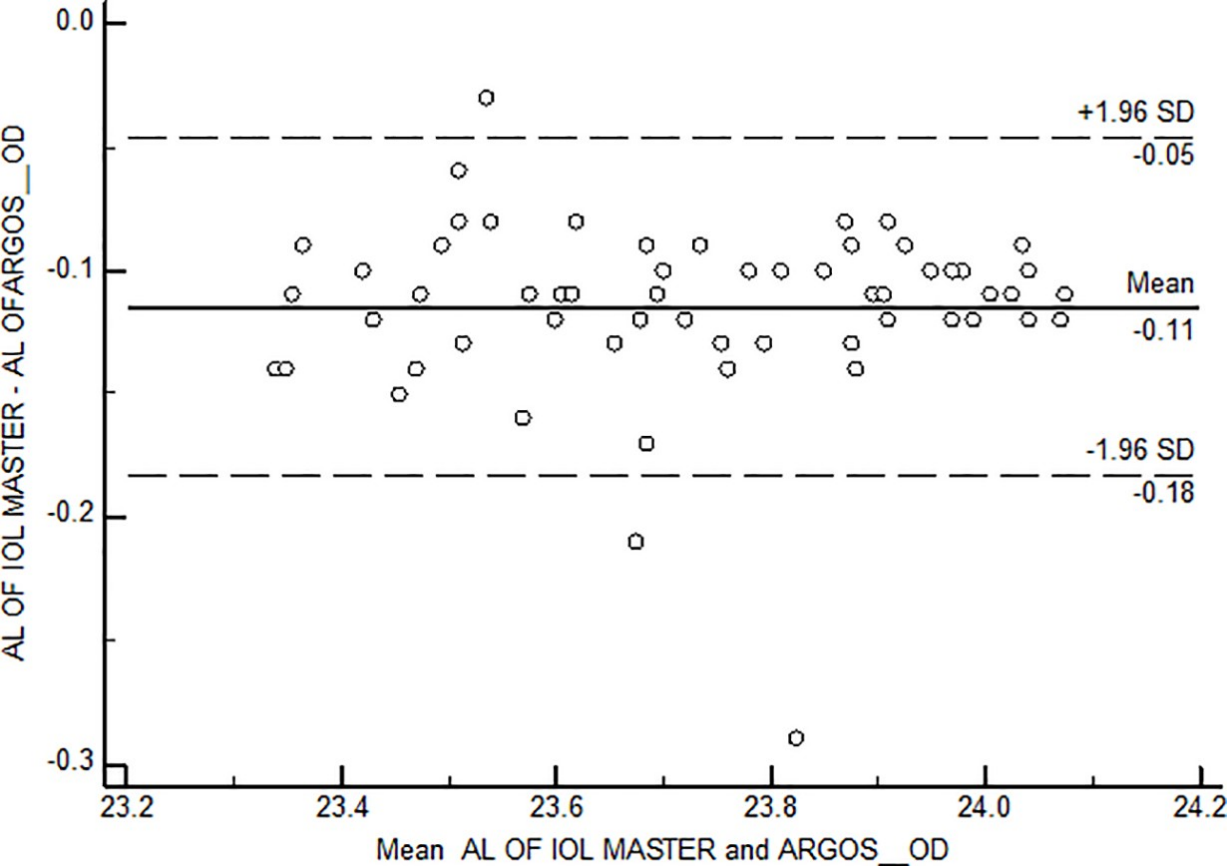
Bland Altman Plot for agreement between axial length measurements among Intermediate axial length group. AL-Axial length; OD–Right eye.

**Fig 8:**
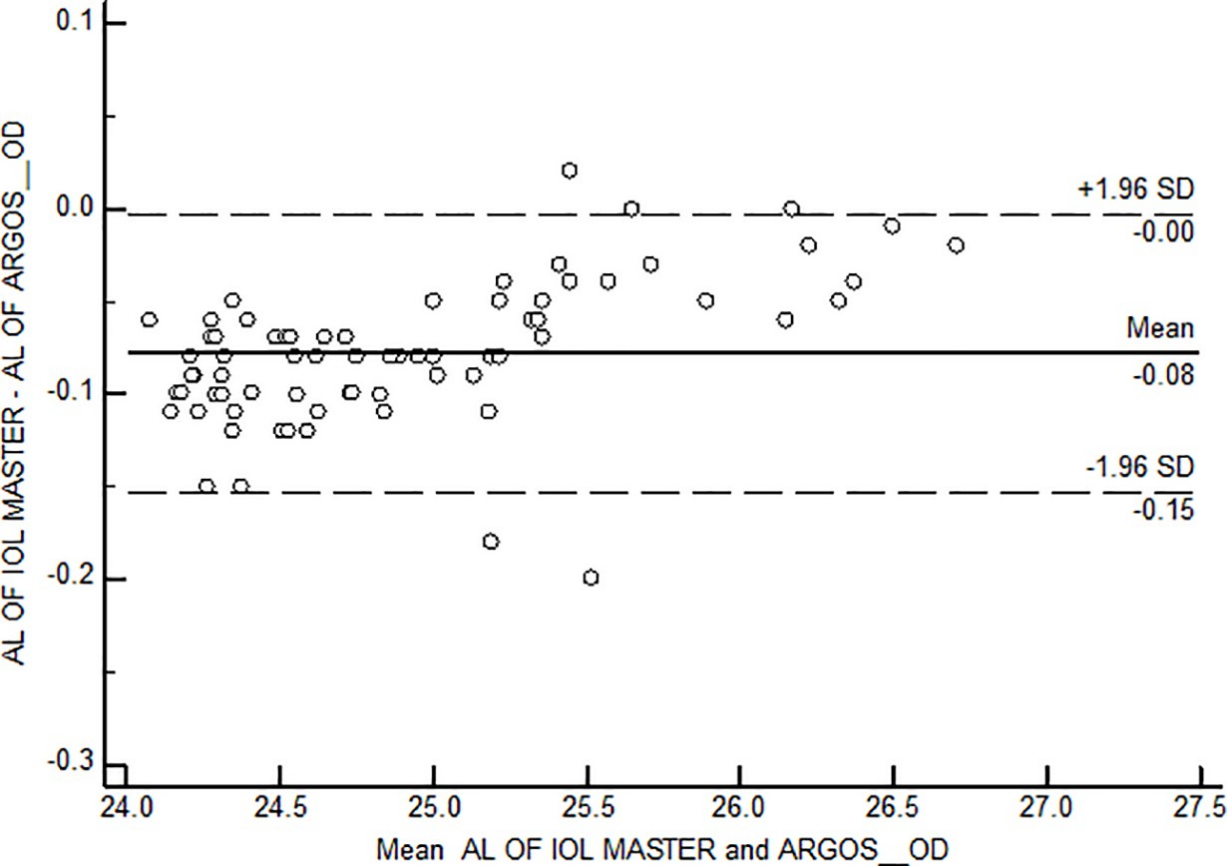
Bland Altman Plot for agreement between axial length measurements among long axial length group. AL-Axial length; OD–Right eye.

Bland Altman plots were constructed for each of the axial length groups; the mean difference was -0.13mm, -0.11mm and -0.08 mm among short, intermediate and long axial lengths (Fig 6, Fig 7 and Fig 8)

## Discussion

The prevalence of myopia is increasing rapidly especially in some of the urban East Asian countries and it is predicted that almost half of the world’s population would become myopic by 2050 [15]. Ocular biometry parameters are essential to understand the process of emmetropization and to assess risk factors for the onset of myopia. When there is a mismatch between these parameters especially axial length and corneal curvature, refractive error sets in.

The ratio of axial length and corneal curvature is identified as a potential risk factor for myopia onset [16] and hence it is essential to incorporate the two major ocular biometry components in to the regular school vision screening programmes to identify children at risk for onset of myopia. Also, to monitor the effectiveness of any control measures to retard progression of myopia the documentation of ocular biometry parameters in children becomes important.

Prior to the advent of non contact biometers, axial length was measured using ultrasound biometry which was essentially a contact procedure. These measurements required expertise and training and the values obtained had an inherent possibility of being inaccurate due to the contact nature of the testing protocol. The limitations of contact biometers have been overcome by the advent of non contact biometers which are easy to operate and the measurements can be obtained relatively within few minutes.

The new swept source (SS–OCT) based anterior segment biometry is advantageous over other non contact biometers due to its ability to acquire high speed three dimensional anterior segment data in few milliseconds along with high lateral and axial resolution [17].PCI based IOLMaster is the current gold standard for non contact biometry measurement and hence the aim of the study was to look at the agreement between these two biometers among children.

There was a strong positive correlation between the mean axial lengths and corneal curvature given by the two biometers in the present study. Some of the previous studies reported the agreement of axial length measurements between SS-OCT and PCI [7, 9, 10, 11]. Shammas et al [7] had looked at the repeatability, reproducibility, and agreement between the SS-OCT based ARGOS and PCI based IOLMaster biometers among adults with cataract and found an overall agreement between the two biometers for axial length and average anterior corneal curvature. Huang et al [9] measured biometry parameters on 65 normal subjects with SS-OCT and PCI based biometers and reported that the repeatability and reproducibility of SS-OCT biometer were good for all biometry parameters and had high agreement between SS-OCT and PCI biometer for most of the biometry parameters. Higashiyama et al [11] compared the axial lengths measured using ARGOS and IOLMaster on 48 eyes, and found that the difference between the axial lengths measured were statistically significant but not clinically significant. They concluded that ARGOS underestimated axial length in longer axial length eyes and overestimated axial length in shorter eyes. Bland Altman plots were constructed to look at the agreement between the two biometers in short, intermediate and long axial lengths. There was an overestimation of axial length by 0.13mm when measured with ARGOS in short eyes and the trend decreased with increase in axial length measurements. There was no underestimation of axial length in longer eyes in the present study. The difference could have been due to the difference in sample size. Nevertheless, the difference was within the clinically acceptable limits of 0.50D as was observed in the previous study. [11]

All of the above mentioned studies were done on adults and to the best of our knowledge this is the first study to look at the agreement of these two non contact biometers among children. Among the pediatric population, a recent study by Chen et al [18] compared axial length, anterior corneal curvature and anterior chamber depth measurements between IOL Master and AL- Scan (Nidek Co.,). The mean difference in AL was close to zero (LoA -0.05 to 0.05) and the mean difference in keratometry was reported to be -0.13D (LoA -0.37 to 0.10). In this study, the mean difference was -0.11 mm (LoA -0.19 to -0.02) for AL and 0.02 D (LoA -0.28 to 0.32) for keratometry measurements. The mean difference and the limits of agreement obtained in this study are comparable to previous literature, and also within the clinically acceptable limits.

Besides axial length and corneal curvature, ARGOS provides Aqueous Depth, Anterior chamber depth, crystalline lens thickness, corneal curvature, central corneal thickness, pupil size and corneal diameter, with a faster acquisition rate that could be beneficial especially when intended to use in pediatric population. ARGOS also facilitates analyzing the data manually and can be used to re-align the spikes when the instrument fails to automatically detect the anterior and posterior margins of the various ocular structures. This is an advantage especially while performing the measurements in children with limited attention span.

The strengths of the study include a large sample size, random sampling in a community set up, and wider range of refractive errors for comparison. The axial length range studied is between 21 to 26.5 mm, and so the results could not be extrapolated to longer axial lengths, this being a possible limitation of the study.

The present study data can be used as a reference for the pediatric axial length measurements. ARGOS SS-OCT based biometer can also be recommended in the pediatric age group due to the speed of acquisition and improved resolution rates. In conclusion, axial length measurements obtained using ARGOS- SS-OCT and IOL Master -PCI based biometers were well within the clinically agreeable limits among pediatric population and is found to be comparable for shorter and intermediate axial length.

## Supporting information

S1 Data (XLSX)
